# A Review of Cross-Species Transmission Mechanisms of Influenza Viruses

**DOI:** 10.3390/vetsci12050447

**Published:** 2025-05-07

**Authors:** Xianfeng Hui, Xiaowei Tian, Shihuan Ding, Ge Gao, Jiyan Cui, Chengguang Zhang, Tiesuo Zhao, Liangwei Duan, Hui Wang

**Affiliations:** 1 Department of Immunology, School of Basic Medical Sciences, Xinxiang Medical University, Xinxiang 453003, China; xianfenghui@163.com (X.H.);; 2 Henan Key Laboratory of Immunology and Targeted Drug, Xinxiang Medical University, Xinxiang 453003, China; 3 Xinxiang Engineering Technology Research Center of Immune Checkpoint Drug for Liver-Intestinal Tumors, Xinxiang Medical University, Xinxiang 453003, China; 4 Department of Pathogenic Biology, School of Basic Medical Sciences, Xinxiang Medical University, Xinxiang 453003, China; 5 National Key Laboratory of Agricultural Microbiology, Huazhong Agricultural University, Wuhan 430070, China; 6 Henan Collaborative Innovation Center of Molecular Diagnosis and Laboratory Medicine, School of Medical Technology, Xinxiang Medical University, Xinxiang 453003, China

**Keywords:** influenza virus, cross-species transmission, mechanisms, One Health

## Abstract

The cross-species transmission of influenza viruses represents a critical link in the pandemic of zoonotic diseases. This mechanism involves multi-level interactions, including viral genetic adaptability, host–receptor compatibility, and ecological drivers. Recent studies have highlighted the essential role of mutations in hemagglutinin and neuraminidase in overcoming host barriers, while elucidating the differences in the distribution of host sialic acid receptors. Furthermore, the “mixer” function of intermediate hosts, such as pigs, plays a significant role in viral redistribution. Advances in high-throughput sequencing and structural biology technologies have gradually resolved key molecular markers and host restriction factors associated with these viruses. However, challenges remain in understanding the dynamic evolutionary patterns of virus–host interaction networks, developing real-time early warning capabilities for cross-species transmission, and formulating broad-spectrum prevention and control strategies. Moving forward, it is essential to integrate multidisciplinary approaches to establish a multi-level defense system, leveraging the ‘One Health’ monitoring network, artificial intelligence prediction models, and new vaccine research and development to address the ongoing threat of cross-species transmission of influenza viruses. This paper systematically reviews the research progress and discusses bottlenecks in this field, providing a theoretical foundation for optimizing future prevention and control strategies.

## Simple Summary

The cross-species transmission of influenza viruses is pivotal in zoonotic pandemics, driven by viral genetic adaptability, host–receptor compatibility, and ecological factors. Key mutations in hemagglutinin and neuraminidase enable host barrier breaches, while intermediate hosts (e.g., pigs) act as viral “mixers” for strain diversification. Advances in sequencing and structural biology have identified critical molecular markers, yet challenges persist in deciphering dynamic virus–host evolution, establishing real-time surveillance, and designing broad-spectrum interventions. Integrating multidisciplinary strategies—including One Health networks and artificial intelligence-driven prediction models—is crucial for building a multi-layered defense system. This review synthesizes current progress and challenges, offering a framework to optimize pandemic preparedness against influenza spillover risks.

## 1. Introduction

Influenza viruses pose a significant threat to both human and animal health, characterized by their ability to cross species barriers and cause epidemics and pandemics [1,2]. The global status of animal influenza has been documented through extensive surveillance, revealing a rich diversity of influenza subtypes across various host species [3]. A comprehensive database compiled up to 2016 identified over 70,000 records of animal influenza events, highlighting the ongoing risk of new subtypes emerging from animal reservoirs, particularly in regions like Asia, North America, and Europe, where the diversity of influenza viruses is greatest [4]. This diversity is a critical factor in the potential for reassortment, which can lead to the emergence of novel strains with pandemic potential [4].

In recent years, significant progress has been made in understanding the mechanisms of cross-species transmission of influenza viruses. Studies have demonstrated that avian influenza viruses (AIVs) possess the ability to adapt to mammalian hosts through specific mutations that enhance their capacity to bind to human-type receptors [5,6]. For instance, the H7N9 avian influenza virus has been identified as dual-receptor tropic, indicating a limited potential for human-to-human transmission, although certain mutations may augment this capability [7,8,9]. Similarly, by analyzing the changes in the receptor-binding specificity of influenza viruses to host receptors, researchers can trace the adaptive evolution of swine influenza viruses, as this specificity is critical for their efficient transmission among pig populations. The mechanisms underlying the interspecies transmission of influenza viruses are complex and involve various factors, including the host’s immune response and the virus’s ability to evade these defenses [10]. Type I interferons (IFNs) play a crucial role in the host’s defense against influenza virus infections, but influenza viruses have evolved strategies to counteract these responses [11,12], complicating the dynamics of transmission. Additionally, the polymerase basic protein 2 (PB2) has been implicated in the adaptation of influenza viruses to new hosts, with specific mutations influencing the virus’s ability to replicate and transmit effectively [13,14].

Among the diverse viral families with cross-species transmission potential, influenza viruses are particularly concerning due to their ability to jump between hosts and generate novel, potentially pandemic strains. While influenza B and C viruses exhibit limited zoonotic potential due to their restricted host ranges—primarily affecting humans and seals—and relatively stable genomic architectures [15,16], influenza A viruses (IAVs) demonstrate unparalleled cross-species versatility. Genomic surveillance has identified IAV infections across 12 mammalian orders, ranging from cetaceans to primates [17], as well as in all major avian taxa [18]. This remarkable host breadth is driven by two primary evolutionary features: first, the virus’s co-evolution with aquatic wild birds, which serve as an ancient and genetically diverse reservoir [18,19]; and second, the capacity for adaptive mutations in the viral hemagglutinin (HA) protein, which enable flexible receptor binding and facilitate interspecies transmission [20]. These properties position IAV as a central model for studying zoonotic emergence and pandemic risk. The pandemic potential of IAVs is also influenced by their genetic diversity and the ability to undergo rapid evolution [21]. Continuous surveillance and research are essential to identify viruses with pandemic potential before they fully adapt to human populations. This includes understanding the ecological factors that facilitate the emergence of new strains, such as the close contact between humans and animals in agricultural settings [22]. Moreover, the role of exposed swine in the transmission of influenza A viruses to humans has been highlighted, with cases often occurring among individuals with close contact to swine [23]. This underscores the importance of biosecurity measures in preventing the spread of influenza viruses across species [24]. In summary, the epidemic status of influenza viruses is marked by their ongoing evolution and potential for cross-species transmission, necessitating robust surveillance and research efforts to mitigate the risks associated with these viruses [25]. Understanding the mechanisms of transmission and adaptation is crucial for developing effective prevention strategies and vaccines to combat future influenza pandemics. We aim to summarize the current knowledge about influenza, including the classification, epidemic status, cross-species dissemination mechanisms, public health importance, and prospects and challenges of cross-species transmission of influenza viruses.

## 2. Classification of Influenza Viruses

The influenza virus is an enveloped negative-strand RNA virus that belongs to the genus Influenza within the Orthomyxoviridae family. Based on differences in the nucleoprotein (NP) and matrix protein (M), the virus can be categorized into four types: A, B, C, and D [26,27,28].

**Figure 1:**
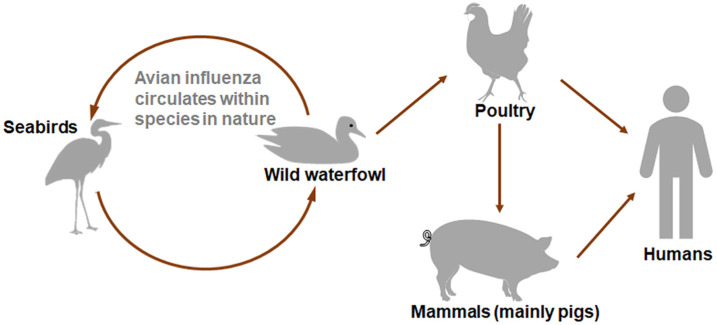
Transmission routes of influenza epidemics.

Among these pathogens, the influenza A virus stands out as the most common threat to both humans and animals, leading to what is commonly known as seasonal flu [16]. Additionally, the influenza A virus was responsible for four significant pandemics: the 1918 Spanish influenza, the 1957 Asian influenza, the 1968 Hong Kong influenza, and the 2009 swine-origin pandemic influenza (2009 pH1N1). It was also the cause of a comparatively milder pandemic, known as the 1977 Russian influenza [29,30,31]. Regarding the origins, development, and ecological aspects of influenza viruses, migratory birds, including shorebirds and waterfowl, are considered major reservoir hosts, offering a diverse array of viral gene segments that can lead to the emergence of new reassortant viruses [18,19,32]. Concurrently, pigs are widely regarded as optimal “mixing vessels” for influenza A viruses due to their susceptibility to both avian- and human-origin strains. This is facilitated by the co-expression of α2,3- and α2,6-linked sialic acid receptors in the porcine respiratory epithelium [33], allowing for co-infection and genetic reassortment. A prominent example is the 2009 H1N1 pandemic virus, which arose through triple reassortment involving avian, human, and swine influenza lineages (Figure 1) [34].

Influenza viruses that circulate among human populations include the influenza B virus (IBV) in addition to IAV [35]. Both of these virus types are included in the formulations of current seasonal influenza vaccines. Historically, IBV has been responsible for approximately 20% of influenza-related hospitalizations each year and can dominate specific influenza seasons, as observed in Europe during 2017/2018 [35]. The severity of illness caused by IBV is similar to that caused by IAV, particularly affecting children and young adults who are most vulnerable to IBV [36,37]. In contrast to IAV, which is typically found in aquatic bird populations, IBV infections are primarily confined to humans, with very few instances of infection reported in seals [15]. However, mice and ferrets can be experimentally infected with IBV, making these animals valuable models for studying human IBV infection and related diseases [38,39]. Due to its restricted host range and a significantly slower mutation rate compared to IAV, circulating IBV strains show less antigenic diversity when compared to H1N1 and H3N2 IAV strains [40,41]. Despite this, since the initial reports in the 1940s, IBV has gradually evolved into two separate lineages—B/Victoria/2/87-like and B/Yamagata/16/88-like—henceforth referred to as the B/Victoria and B/Yamagata lineages, respectively, which are further subdivided into distinct antigenic clades [36,42].

Unlike influenza A and B viruses that infect humans and cause severe diseases in seasonal epidemics, the influenza C virus (ICV) is a ubiquitous childhood pathogen typically causing mild respiratory symptoms [43,44]. Additionally, ICVs can infect various animals, including pigs, dogs, and cattle [45]. However, ICVs are less likely to present a significant threat to human health [46,47]. Influenza D virus (IDV) is considerably rarer, with no documented cases of human infection. IDVs primarily infect a limited range of animals, including pigs and cattle, and have a minimal impact on public health [48]. Currently, there are no recognized subtypes of influenza C and D viruses.

## 3. Epidemic Status of Influenza A Virus

Influenza viruses pose significant threats to both agricultural sectors and human health, primarily due to their ability to infect a wide range of hosts, including birds, pigs, and humans [21]. The epidemiology of these viruses is complex, characterized by frequent interspecies transmission and reassortment, which can lead to the emergence of novel strains with pandemic potential [49,50].

**Figure 2:**
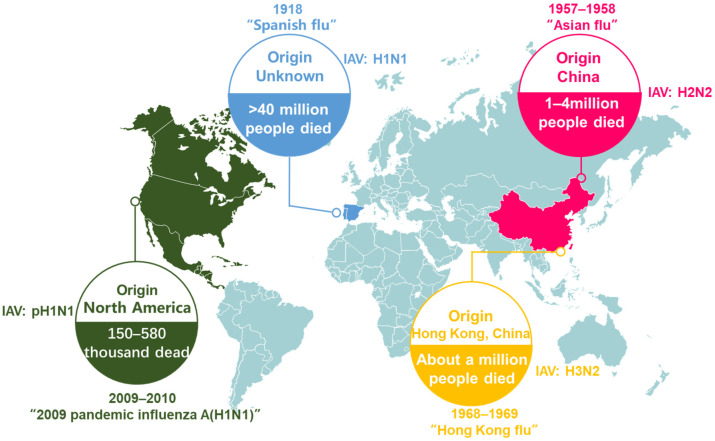
Four global flu pandemics recorded since the early twentieth century.

It is estimated that seasonal infections caused by the influenza virus lead to approximately 250,000 to 500,000 deaths each year globally [51]. In healthy adults, seasonal influenza infections are typically self-limiting and mainly confined to the upper respiratory system; however, these infections can be severe in children and the elderly, potentially resulting in viral pneumonia. Besides humans, the A strains of the influenza virus have the capability to infect a variety of host species, including waterfowl, pigs, domesticated birds, and seals. Consequently, influenza A viruses present in zoonotic reservoirs have sporadically led to widespread infection and even pandemics among humans [21]. The most recent four pandemics related to influenza—namely, the 1918 H1N1 Spanish flu [52,53], the 1957 H2N2 Asian flu [54,55], the 1968 H3N2 Hong Kong flu [56], and the 2009 H1N1 [34,57]—were due to the transmission of influenza A virus from zoonotic reservoirs to humans (Figure 2). Additionally, strains of the influenza A virus, such as H5N1, H7N7, and H7N9, have successfully crossed the species barrier from domestic poultry to induce lethal infections in humans [58,59,60].

In agriculture, particularly in the poultry and swine industries, IAV has been responsible for severe economic losses [61,62]. Avian influenza viruses can be divided into highly pathogenic avian influenza (HPAI) viruses and low pathogenic avian influenza (LPAI) viruses [63]. Highly pathogenic avian influenza viruses are limited to the H5 and H7 subtypes, which cause severe illness and high mortality rates in gallinaceous species. In contrast, low pathogenic avian influenza (LPAI) viruses are typically maintained in wild aquatic birds and can be transmitted to domestic poultry species, often resulting in subclinical infections or mild respiratory diseases [64].

The H5N1, a highly pathogenic avian influenza (HPAI) subtype of influenza A virus, was initially identified in domestic geese in Guangdong, China, in 1996 and has since been responsible for numerous outbreaks among bird populations [65]. Starting in 2020, a variant from the evolutionary lineage 2.3.4.4b of the H5 avian influenza virus has devastated many regions across Africa, Asia, and Europe, resulting in unprecedented mortality rates among wild birds and poultry. The virus reached North America in 2021 and spread to Central and South America in 2022 [66]. In 2022, 67 nations across five continents reported occurrences of H5N1 highly pathogenic avian influenza (HPAI) in both poultry and wild bird populations to the World Organization for Animal Health. The death or culling of over 131 million poultry was recorded on impacted farms and in communities. By 2023, as the virus further propagated, outbreaks were noted in an additional 14 countries, primarily in the Americas. In several instances of reported mass fatalities among wild birds, the causative agent has been confirmed as the influenza A H5N1 clade 2.3.4.4b virus [67].

Avian influenza viruses are widely recognized for their transmission among birds; however, there has been a notable increase in the detection of H5N1 avian influenza viruses in mammals. In April 2024, a dairy farm worker in Texas was diagnosed with H5N1 virus infection after contact with infected cows. This case represents the first documented instance of cattle transmission of the virus, with the primary symptom reported by the patient being conjunctivitis. As of 17 January 2025, the United States has confirmed 67 cases of highly pathogenic H5N1 virus infection, the majority of which are associated with contact with cattle [68]. Outside the United States, more than 950 cases of H5N1 bird flu have been reported to the World Health Organization. Notably, the Louisiana Department of Health in the United States reported on 6 January 2025 the first U.S. death from a human infection with the H5N1 HPAI virus (www.cdc.gov/media, accessed on 1 April 2025). Dr. James Lawler, director of the Center for Global Health Security at the University of Nebraska, said the latest changes remind us that “the more widely a virus spreads, especially infecting humans and other mammals, the higher the risk of the virus mutating and adapting to human disease and transmission. That puts all of us at risk”.

## 4. Cross-Species Transmission Mechanism

As mentioned above, cross-species transmission of influenza viruses puts humans at risk. Current research on the cross-species transmission of influenza viruses has highlighted the complex dynamics involved in the adaptation and spread of these viruses among different hosts. The mechanisms underlying this cross-species transmission are multifaceted and involve both viral and host factors. One of the key findings in recent studies is the crucial role of receptor binding specificity in the transmission of influenza A viruses (IAVs). These viruses attach to sialic acid (SA) residues on the surface of host cells, and the type of linkage—either α2,3 or α2,6—plays a pivotal role in determining host susceptibility [10,69]. Avian influenza viruses typically recognize α2,3-linked sialic acids, which are abundant in the intestinal and respiratory tracts of birds, whereas human-adapted strains preferentially bind to α2,6-linked sialic acids, predominantly expressed in the human upper respiratory tract [70,71]. This difference in receptor binding preference represents a major barrier to interspecies transmission. However, accumulating evidence indicates that specific mutations in the viral hemagglutinin (HA) protein, particularly at residues 226 and 228 (H3 numbering), can shift binding specificity from α2,3- to α2,6-linked sialic acids. Notably, substitutions such as Q226L and G228S enhance the virus’s ability to recognize and infect human epithelial cells, thereby facilitating more efficient replication and increasing the potential for human-to-human transmission [72,73,74]. These mutations have been identified in multiple zoonotic strains, including the H2N2 and H3N2 subtypes responsible for past influenza pandemics, highlighting their critical role in host adaptation and cross-species transmission.

Furthermore, researchers conducted surface plasmon resonance (SPR) experiments to detect the virus-encoded hemagglutinin (HA) protein. They discovered that the HA of the bovine H5N1 influenza virus preferentially binds to avian α2-3 sialic acid receptors, while also demonstrating a weak binding affinity to human α2-6 sialic acid receptors. Subsequent immunohistochemical staining indicated that the H5N1 HA protein exhibits strong binding to bovine lung and breast tissues, correlating with the observed clinical symptoms. In contrast, the HA proteins of common human seasonal influenza viruses (H1N1 and H3N2) do not effectively bind to the corresponding tissues in cattle [75]. Moreover, the bovine H5N1 HA protein shows significant binding to human conjunctiva, trachea, lung, and breast tissues, whereas the HA proteins of H1N1 and H3N2 viruses demonstrate significant binding only to human tracheal tissue. This finding provides a comprehensive explanation for the molecular mechanism underlying the association of the bovine H5N1 virus with human conjunctivitis and underscores its distinct tissue tropism compared to seasonal influenza viruses.

The evolutionary dynamics of influenza viruses also play a critical role in cross-species transmission. Over the past century, the emergence of an influenza A virus featuring a new NP gene segment has taken place on just two occasions, each resulting in pandemics: the first in 1918 with the “Spanish” H1N1, which originated from an avian virus, and the second in 2009 with pH1N1, a reassortant virus made up of gene segments from two swine influenza viruses that formed a stable lineage in the human population [34,76,77]. Studies have demonstrated that the introduction of specific mutations, such as the NP-R351K mutation, can significantly enhance the transmissibility of swine influenza viruses. This particular mutation increases viral transmission through multiple mechanisms: enhanced polymerase activity, improved ribonucleoprotein (RNP) assembly, greater thermostability, and more efficient nuclear import. Together, these effects elevate transmission efficiency by 35–40% in mammalian hosts, demonstrating how even minor genetic modifications can substantially impact viral spread [13,78].

In addition to viral factors, host characteristics are also crucial in determining the success of cross-species transmission [79]. The composition of the upper respiratory tract (URT) flora, for example, can influence the susceptibility of hosts to IAV infection [80]. Recent research has utilized mouse models to explore how variations in the URT environment affect viral transmission. It was found that infant mice, which exhibit different sialic acid profiles compared to adults, support more efficient transmission of IAV among littermates [80,81,82,83]. This suggests that age and the specific composition of the URT can significantly impact the dynamics of viral spread. Additionally, host factors such as immune status and underlying health conditions can alter the efficiency of influenza virus transmission. For example, children and immunocompromised individuals often exhibit higher viral loads and prolonged periods of viral shedding, thereby increasing the risk of transmission [84,85]. Furthermore, host immune responses, including innate antiviral defenses like interferon signaling, can either limit or facilitate viral propagation depending on their timing and strength [86,87].

Moreover, environmental changes play a crucial role in shaping the transmission dynamics of influenza viruses. Key factors such as temperature, humidity, and seasonal variation directly influence viral stability and infectivity. In temperate regions, influenza activity peaks during colder months, where low temperatures and reduced absolute humidity enhance the stability of influenza virions in aerosols and on surfaces, thereby promoting airborne transmission. Conversely, in tropical regions, transmission patterns are often associated with the rainy season and increased indoor crowding [88,89]. Furthermore, environmental conditions can indirectly affect human behavior, such as increased indoor activity during cold or wet weather, which facilitates close contact and enhances the likelihood of person-to-person transmission [90,91]. Climate change may also alter these patterns by shifting the seasonality of influenza or changing the geographical distribution of outbreaks.

Additionally, environmental factors, such as close contact between hosts and the presence of contaminated surfaces, can facilitate transmission [92]. For instance, the housing conditions of swine farms have been shown to enhance the likelihood of avian viruses spilling over into swine populations, which can then serve as intermediaries for transmission to humans [93].

## 5. Human Activities and Cross-Species Transmission of Influenza Viruses

**Figure 3:**
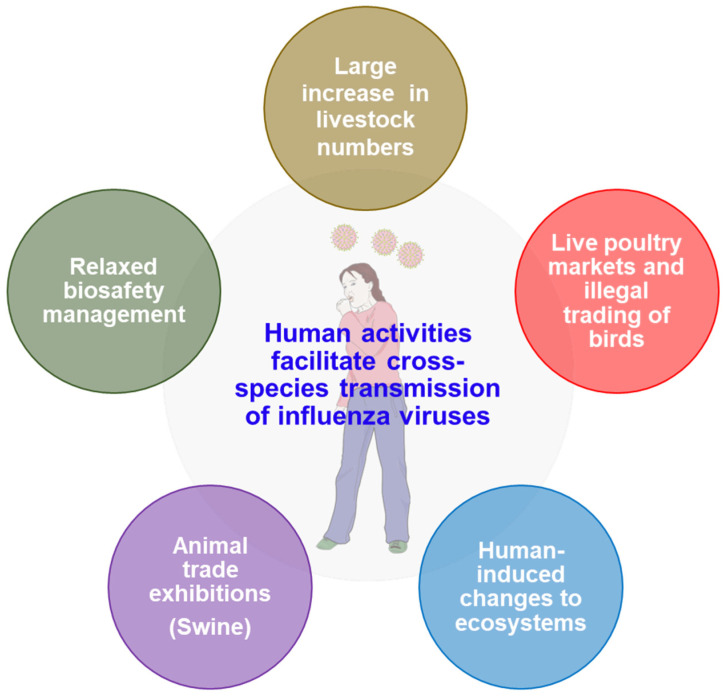
Human activities facilitate cross-species transmission of influenza viruses.

Human activities significantly influence the dynamics of cross-species transmission of influenza viruses, primarily through alterations in animal populations, environmental changes, and increased interactions between humans and wildlife [94,95,96,97]. The interplay between these factors creates a complex landscape that facilitates the emergence and spread of zoonotic influenza viruses (Figure 3).

One of the most critical aspects of human impact is the increase in livestock populations, particularly poultry and swine, which has been linked to a rise in zoonotic influenza infections [92,98]. The industrialization of agriculture has led to higher densities of animals in confined spaces, creating ideal conditions for the transmission of viruses among species. For instance, the rise in poultry and swine populations since the industrial revolution has been associated with an increased frequency of zoonotic influenza virus infections in humans, highlighting the direct correlation between human agricultural practices and the risk of influenza outbreaks [92,99,100]. Moreover, specific human activities, such as illegal trading of birds, live poultry markets, and practices like cockfighting [101,102], have been identified as significant contributors to the spread of highly pathogenic avian influenza (HPAI) viruses, such as H5N1 [80,103]. These activities not only increase the likelihood of direct contact between infected animals and humans but also facilitate the mixing of different virus strains, which can lead to the emergence of new variants capable of infecting humans [50,103,104,105].

Environmental factors also play a crucial role in cross-species transmission. Human-induced changes to ecosystems, such as deforestation, urbanization, and climate change, can disrupt the natural habitats of wildlife, forcing animals into closer contact with human populations [96,106]. This increased interaction raises the risk of zoonotic spillover events, where viruses jump from animals to humans. For example, the movement of migratory birds, which can carry influenza viruses, is influenced by environmental changes, potentially leading to new transmission pathways [107,108].

Moreover, the exhibition swine industry exemplifies how human management practices can facilitate the rapid dissemination of influenza viruses. Close and prolonged exposure to exhibition swine has led to variant influenza infections in humans, particularly among swine exhibitors [24,49,109]. The concurrent detection of genetically identical influenza A viruses from exhibition swine across different states highlights the mobility of this population and the potential for rapid virus spread [24,110,111]. The relaxed biosecurity in these settings further exacerbates the risk of transmission, allowing for the movement of pathogens across large geographic areas.

## 6. Strategies and Prospects for Human Response to Interspecies Transmission of Influenza Virus

Cross-species transmission of influenza viruses poses significant challenges to public health, particularly due to the potential for novel strains to emerge that can infect humans and cause pandemics. Addressing this issue requires a multifaceted approach that includes surveillance, biosecurity measures, vaccination strategies, and research into the mechanisms of viral adaptation. One of the primary strategies for managing cross-species transmission is the implementation of robust surveillance systems. Continuous monitoring of influenza viruses in both human and animal populations is essential for the early detection of emerging strains. This includes surveillance of wildlife, domestic animals, and humans, especially in areas where interactions between humans and animals are common. For example, monitoring pig populations is crucial, as pigs can act as mixing vessels for avian and human influenza viruses. This interaction can lead to the emergence of reassortant strains that may pose a risk of pandemics [112,113]. In addition to surveillance, enhancing biosecurity practices in agricultural settings is crucial. This involves improving the separation of different animal species, such as pigs and poultry, to reduce the risk of interspecies transmission. Farmers should adopt better biosecurity measures, such as controlling animal movement, maintaining clean facilities, and minimizing contact between livestock and wild birds [112]. Furthermore, public health strategies should be developed to educate farmers and traders about the risks associated with influenza transmission and the importance of implementing these biosecurity measures.

Vaccination is another vital component in the fight against influenza viruses. Current seasonal influenza vaccines are effective against strains that are closely related to those included in the vaccine; however, the antigenic variability of influenza viruses necessitates annual updates to the vaccine composition [114]. The development of a universal influenza vaccine that can provide broad protection against multiple strains is a promising area of research. Such a vaccine would target conserved viral epitopes, potentially offering protection against a wide range of influenza viruses, including those that have not yet emerged [114].

While human vaccination remains a cornerstone of influenza prevention, controlling influenza viruses at the animal level is equally crucial, particularly in species that serve as reservoirs or intermediate hosts, such as swine and poultry [49,115]. These animals often act as “mixing vessels”, where avian, swine, and human influenza strains can reassort, giving rise to novel variants with zoonotic or even pandemic potential [49,116]. Vaccination in these animal populations not only reduces the burden of disease within the species but also significantly decreases viral shedding and transmission risk to humans. For instance, targeted immunization programs in swine have been shown to mitigate the risk of variant influenza virus emergence at agricultural fairs [117,118]. Similarly, vaccination in poultry is a critical control strategy in limiting the spread of highly pathogenic avian influenza and preventing spillover to human populations. Therefore, integrating animal vaccination into a One Health approach is essential for comprehensive influenza control and pandemic preparedness.

Research into the molecular mechanisms of influenza virus adaptation is also essential for understanding cross-species transmission. Studies have shown that specific mutations in viral proteins, such as the polymerase basic protein 2 (PB2), play a significant role in the virus’s ability to adapt to new hosts [119]. Understanding these mechanisms can inform the development of targeted interventions to prevent the emergence of new strains. For example, identifying mutations that confer increased transmissibility in mammals can help prioritize surveillance efforts for specific viral variants [83,113,120].

**Figure 4:**
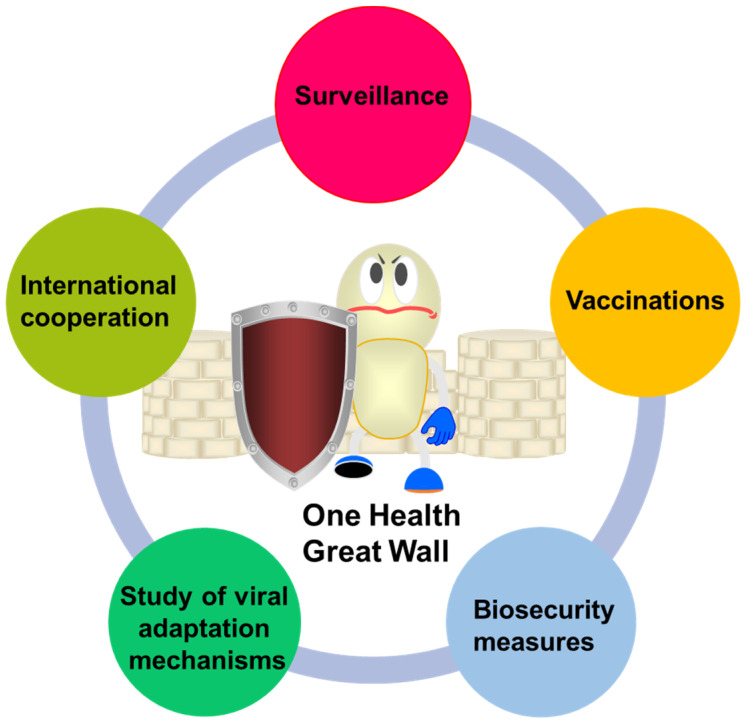
The Great Wall of One Health for all mankind.

Moreover, the role of environmental factors in facilitating cross-species transmission should not be overlooked. Ecological changes, such as habitat destruction and climate change, can alter the dynamics of host interactions and increase the likelihood of spillover events. Addressing these environmental factors through conservation efforts and sustainable agricultural practices can help mitigate the risk of influenza virus transmission [121]. Finally, international collaboration among public health officials, veterinarians, and researchers is crucial for effectively managing the risks associated with cross-species transmission. Sharing data and resources can enhance the global response to influenza outbreaks and improve preparedness for potential pandemics. Collaborative efforts can also facilitate the development of comprehensive strategies that integrate animal and human health, often referred to as a One Health approach [92]. In summary, addressing the cross-species transmission of influenza viruses necessitates a multifaceted approach that includes surveillance, biosecurity measures, vaccination strategies, research into viral adaptation, and international collaboration (Figure 4). By employing these strategies, public health officials can more effectively manage the risks associated with influenza viruses and diminish the likelihood of future pandemics.

## 7. Conclusions

Cross-species transmission of influenza viruses results from the intricate interplay between viral evolution, host adaptability, and ecological environments. This phenomenon poses significant challenges to global public health security. The essence of cross-species transmission lies in the virus’s pursuit of survival advantages within dynamic interactions. Despite notable advancements in understanding the underlying mechanisms and developing intervention strategies, the unpredictability of viral mutations, the complexity of ecosystems, and the impacts of human activities continue to elevate the risk of future pandemics. To effectively address this issue, it is essential to foster continuous innovation in basic research, promote global resource sharing for monitoring, and enhance public health literacy. These efforts will empower us to take the initiative in the ongoing struggle between “viral evolution” and “human defense”.

Recent technical advancements have enabled researchers to investigate influenza virus infections across various biological levels, ranging from cellular interactions to whole organisms. This progress has contributed to a deeper understanding of the virus’s biology, disease severity, and the immune response. However, despite these advancements, our knowledge remains incomplete regarding the viral properties that could potentially lead to pandemics. Future research should focus on comprehensive mutagenesis studies, cataloging receptor binding, and expanding surveillance in poultry and pigs, particularly in regions such as Africa and South America. These efforts aim to develop robust computational tools and novel animal models to enhance our understanding of transmission dynamics and inform vaccine development strategies [120,122]. It is important that we strive to advance human research proactively in anticipation of viral mutations, rather than relying solely on passive prevention and control measures after a pandemic has emerged.

In summary, the current research status on the cross-species transmission of influenza viruses reveals a dynamic interplay between viral genetics, host factors, and environmental conditions. Understanding these mechanisms is crucial for predicting and mitigating the risks associated with influenza pandemics.

## Data Availability

No new data were created or analyzed in this study. Data sharing is not applicable to this article.
